# Effect of Edible Onion (*Allium cepa* L.) Film on Quality, Sensory Properties and Shelf Life of Beef Burger Patties

**DOI:** 10.3390/molecules26237202

**Published:** 2021-11-27

**Authors:** Kallyne Sousa Soares, Marthyna Pessoa Souza, Edson C. Silva-Filho, Hernane Silva Barud, Clóvis Augusto Ribeiro, Diógenes Dias Santos, Karla Nayalle Souza Rocha, José Fabio Paulino de Moura, Ronaldo Lopes Oliveira, Leilson Rocha Bezerra

**Affiliations:** 1 Department of Animal Science, Animal Health and Science Graduate, Federal University of Campina Grande, Patos 58798119, Paraiba, Brazil; marthynapessoa@yahoo.com.br (M.P.S.); jose.fabio@ufcg.edu.br (J.F.P.d.M.); 2 Chemistry Department, Laboratory of Advanced Materials, Federal University of Piauí (UFPI), Teresina 64049550, Piauí, Brazil; edsonfilho@ufpi.edu.br; 3 Department of Biotechnology, University Center of Araraquara (UNIARA), Araraquara 14801320, São Paulo, Brazil; hernane.barud@gmail.com; 4 Chemical Institute, São Paulo State University (UNESP), Araraquara 14800900, São Paulo, Brazil; clovis.ribeiro@unesp.br; 5 BioSmart Nano Technology, Araraquara 14808162, São Paulo, Brazil; digignes@gmail.com; 6 Health Department, Federal Institute of Education, Science and Technology of Paraiba/Campus Patos, Patos 58700000, Paraiba, Brazil; karlanayalle@ufpi.edu.br; 7 Department of Animal Science, Federal University of Bahia, Salvador 40170110, Bahia, Brazil; rornaldooliveira@ufba.br

**Keywords:** edible coating, beef burgers patties, antimicrobial, lipid oxidation, texture

## Abstract

The production of edible film from onion (*Allium cepa* L.) to be applied as packaging is attractive, due to its chemical properties and biodegradable characteristics. Thus, we tested the hypothesis that edible onion film can positively influence the sensory properties, quality and increasing shelf life of beef burgers patties. The experiment was designed in a 4 × 2 factorial scheme, with two treatments (beef burgers patties with or without edible onion film) at an interval of four storage times (0, 3, 6 and 9 days) at 4 °C. The uncoated burger patties (control) suffered the most intense color modifications during the storage (*p* < 0.05). The luminosity index was higher (*p* < 0.05) in the control at all storage times, except at day 6, and redness, yellowness and chrome were higher (*p* < 0.05) in the edible onion film patties at all storage times. The pH of the beef burger patties was lower (*p* < 0.05) at all storage times when the edible onion film was applied. For the texture profile, only the chewiness was affected, as the inclusion of the edible onion film improved the chewing of the beef burgers patties over the storage time (*p* < 0.05). Additionally, there was an inhibition of the microbial growth of mesophiles and psychrophiles with the application of the edible onion film in beef burgers patties. The use of edible onion film improved the perception of panelists for the variables texture, color, flavor, odor and overall appearance, and increased the preference of panelists. The edible onion film is recommended for preserving beef burgers patties, as it delays the proliferation of unwanted microorganisms, stabilizes and improves the color parameters and sensory attributes, and increases the overall acceptance of the consumer.

## 1. Introduction

Beef is a food with high nutritional value and is one of the most consumed protein sources in the world and therefore deserves special attention in terms of its preservation and consumption [1]. However, due to its particularities, meat is subject to changes due to chemical, physical and microbiological reactions. Proteolyze and lipid peroxidation can be caused by natural agents such as oxygen, hydrolytic enzymes present in meat and other substances that are produced by the action of microorganisms [2].

Thus, beef becomes an excellent culture medium for microbial growth, whether due to favorable intrinsic factors, such as: chemical composition, high water activity, and pH decline rate; or by extrinsic factors, such as: humidity, temperature and chemical composition of the atmosphere [3]. These factors, together, can change the natural microbiota of meat and contribute to the development of pathogenic and deteriorating microorganisms, with environmental temperature being the most important extrinsic factor that determines microbial multiplication [4].

Another important factor to be considered is the processing of meat into meat products, such as the beef burger patties, which does not significantly modify the original nutritional qualities, but attributes organoleptic characteristics such as color, flavor and aroma, characteristic of each process [5]. In addition, it adds value with the use of unused cuts for fresh consumption, generating greater alternatives for its sale. This enables the development of the industrialization of derived products, contributing to the generation of jobs and increasing the income and offer of commercially available products, in addition to contributing to the preservation of the product.

In this aspect of preservation, this is one of the main functions of food packaging systems, as it separates food from the surrounding environment, reducing the interaction with spoilage factors (such as microorganisms, water vapor, oxygen and unpleasant flavors) and preventing losses of desirable compounds (for example, volatile flavorings), thus extending the shelf life of foods [6]. In addition, beef burgers are more prone to microbial contamination due to the mincing step and greater exposure to oxygen [4,5].

The application of new technologies to improve quality foods and longer commercial time, or shelf life, has been increasing remarkably, in view of the high potential offered by alternative techniques, often combined with traditional methods in terms of preservation. The application of new technologies to prolong the shelf life and improve food sensory quality has been increasing remarkably. In the meat industry, alternative techniques often combined with traditional preservation methods are able to reduce undesirable biochemical effects, such as reducing oxidative processes and microorganism contamination [7].

In the new global economy, petroleum-derived polymers used in packaging applications have become a central issue as their value chains currently involve substantial drawbacks. Although most of these materials have attractive characteristics (e.g., low cost, adequate mechanical properties and processability), their continuous and extensive disposal has raised considerable concerns about their deleterious effects on the environment [8,9,10]. In this scenario, edible and degradable films stand out as an excellent alternative for replacing their synthetic equivalents. With regard to food packaging, edible films can play passive or active roles, depending on the food product itself [9]. Among the non-passive functions are the unique sensory, nutritional and antioxidant characteristics when the edible coatings are composed of fruits and vegetables [6,9].

Edible films can be classified as a primary packaging, which is in direct contact with the product, and it is also necessary to use a secondary, external packaging to protect the food from external contamination and, in some cases, from contact with [10]. Since they are in direct contact with food, edible films must also meet specific regulatory requirements, and for that, the materials used in their formulations must be non-toxic and safe for human consumption. Therefore, they must be thoroughly studied, as they can transfer constituents to food that are not always detected by analytical chemistry, resulting in low human exposures [8].

More than 35 plant species have been used as primary ingredients in the production of edible films [6,11,12]. According to this promising strategy to manufacture innovative packaging without synthetic polymers, onion (*Allium cepa* L.) is a potential source of edible films for applications related to biodegradable packaging [13]. *Allium cepa* L. has several phytonutrients such as: flavonoids, fructooligosaccharides (FOS) and thiosulfide and other sulfur compounds, recognized as important elements of high nutritional value [14]. Quercetin is the main flavonoid present in onion [15], which has several properties, including antioxidant and antimicrobial activity [16]. Films obtained from onion bulbs are promising bioactive sources and have good mechanical and water vapor barrier properties [17]. Furthermore, mutagenicity and cytotoxicity tests demonstrate that these biomaterials are harmless, supporting at the first level of evidence, their safety potential for use as an edible coating by the food industry [18]. Thus, considering the antioxidant properties of onion, we hypothesized that edible packaging manufactured from its pulp may increase the shelf life of perishable foods such as beef burger patties. The objective of this study was to evaluate the effect of edible onion film on the preservation of beef burger patties.

## 2. Results

There was no interaction (*p* > 0.05) between storage time and application of the edible onion film for the variables of color and physicochemical composition of the beef burger patties and, therefore, the factors will be discussed separately as a function of each time.

The color parameters L*, a*, b* and saturation index or Chrome (C*) of the burger patties were affected (*p* < 0.05) by onion film application at storage time (Table 1). Luminosity was higher (*p* < 0.05) in the control at all storage times, except at day 6, and redness, yellowness and chrome were higher (*p* < 0.05) in edible onion film at all storage times. The uncoated burger patties (control) suffered the most intense color modifications during the storage. However, all color parameters decreased slightly from 3rd day onwards (*p* < 0.05).

It was observed that the application of edible onion film in beef burger patties promoted lower pH values (*p* < 0.05) compared to the control treatment at days 3, 6 and 9 (Table 2). Over the storage time, the pH values of the edible onion film samples decreased significantly (*p* < 0.05) between day 3 and 6, whereas the control samples increased the pH values at days 3 and 6 and decreased at 9 days.

The water holding capacity (WHC) of all beef burger patties decreased as the storage time increased. However, it was observed that the beef burger patties coated with the edible onion film were the ones with the lowest values of WHC and the highest rates of cooking loss (CL) at the end of 9 days compared to the control samples.

There was no significant difference (*p* > 0.05) between treatments regarding the amount of iron as well as for the lipid oxidation values. However, the quantification of heme iron content present in the beef burger patties showed a gradual decrease in the amount of Fe over the days in both treatments, whereas, through the analysis of lipid oxidation. evaluated through the measurement of TBARS values. It showed an increase in malonoaldehyde values also occurred in both treatments over the storage period.

Regarding the texture parameter indices there was no effect of the edible onion film in beef burger patties (*p* > 0.05) for the hardness, cohesiveness and springiness variables (Table 3). However, the chewability was better with the application of edible onion film in beef burger patties compared to the control treatment, especially between time 0 and 9 days. It was observed that, over the storage time, the springiness of the edible onion film burgers decreased significantly and, in contrast. the beef burger patties from the control treatment showed an increase (*p* > 0.05) degree of springiness as the days of storage increased.

**Figure 1:**
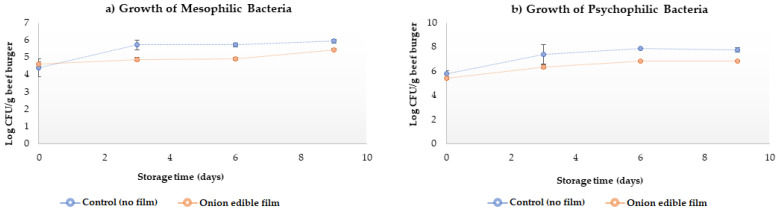
Total aerobic bacteria count (log_10_ CFU) in bovine beef burger patties uncoated (control) and coated with edible onion performed at different storage times (4 °C). Mesophiles (**a**) and psychrophiles (**b**). Each data point represents the mean, and the error bars are the standard deviation (*n* = 3).

There was a greater (*p* < 0.05) growth of mesophilic bacteria in the control treatments when compared to the beef burger patties preserved with the edible onion film from 3 days and persisted until the end of the shelf life (Figure 1a). Regarding the growth of psychrophilic microorganisms. It was observed that the beef burgers patties without film (control) also had higher microbial growth (*p* < 0.05) than the treatment in which the beef burger patties were preserved with edible onion film from the 6th day of preservation (Figure 1b).

**Figure 2:**
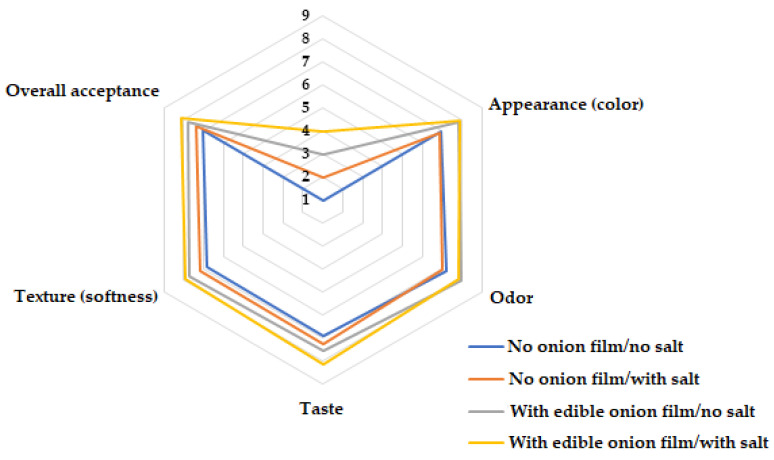
Impression of consumers consuming beef burger patties uncoated (control) and coated with edible onion from a sensory panel in a nine-point hedonic scale (ranging between: “1 disliked it extremely” and “9 liked it extremely”).

The preservation of beef burger patties conserved with edible onion film (with and without salt) was better evaluated by consumers and had the highest scores, with a significant difference (*p* < 0.05) for all analyzed attributes (texture, flavor, color, aroma and overall acceptance) compared to treatments without using the coating (Figure 2).

**Figure 3:**
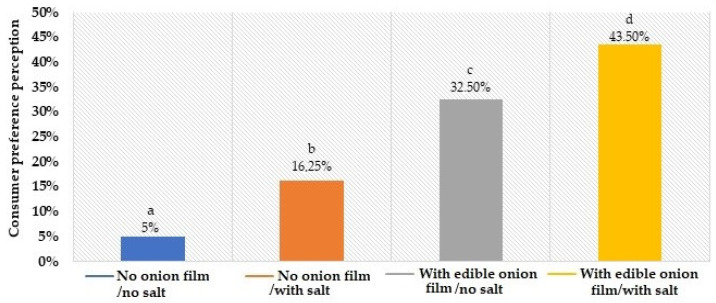
Consumer preference perception of beef burger patties coated or not with edible onion from a sensory panel (means followed by different lowercase letters in the lines differ by Fisher’s LSD test at *p* < 0.05).

The preference ranking (Figure 3) demonstrated that the panelists presented a higher acceptance (*p* < 0.05) by beef burger patties with edible onion film and salt, followed by the beef burger patties with edible onion film and without salt (32.5%). Then beef burger patties without edible onion film and with salt (16.25%) and the lowest acceptance for beef burger patties without edible onion film and without salt (5%).

## 3. Discussion

Color indexes of an edible coating can change the overall appearance of food, since coating color may vary depending on the type of material used for their production. Moreover, in relation to meat, the typical form of myoglobin, the main protein responsible for meat color, associated with low oxygen concentration (deoxymyoglobin—with edible coating) or with oxygenation (oxymyoglobin—without edible coating) can influence meat purchasing decisions [19], so it was important to compare the color of the meat with and without an edible coating [20].

The a*, or redness values, of burger patties presented better stabilization with the application of the edible onion film, possibly caused by changes in the meat structure related to the highly oxidizing conditions [20]. The committed structural and conformational stability of proteins by oxidative damage may result in rupture of the peptide sequence, interactions (protein–protein such as formation or polymerization of aggregates) and modification of the amino acid chains. These modifications caused by oxidative process may alter protein function and its structure. From the most relevant chemical modifications, the formation of protein cross-links and protein carbonylation have been associated with the muscle protein functionality losses and modifications of meat attributes as color, flavor and texture [19,20,21,22]. Also, the maintenance of exudates in the coated meat (Table 2) darkens the color. The a* index of uncoated (control) beef burger patties showed an intense decrease during storage, which decreased slightly in beef burger patties coated with the edible onion film. Meat pigment, in the absence of oxygen, is in the form of deoxy or reduced Mb, which has a purple-red color. On air exposure, the pigment is oxygenated to form MbO2, conferring a bright red color to the meat [20,21]. The coating slowed down the oxygenation process, therefore, instead of reaching the maximum a* value after the first days of blooming due to MbO2 formation, this maximum value is reached at approximately 7 days, decreasing thereafter. This same pattern was observed to b* or yellowness values.

The b* color index (yellow) reflects the amount of fat present in the beef burger patties. Thus, the tendency to decrease these values in the control (uncoated) is related to the process of lipid oxidation present in beef that generates α- and β-aldehydes (secondary products of lipid oxidation) reducing the stability of myoglobin redox [22,23,24]. Onions have been recognized as an essential and valuable source of phytonutrients. as flavonoids, fructooligosaccharides and thiosulfinates and other sulfur compounds [25,26]. In chemical composition, onion represents one of the most common sources of flavonoids in the human diet [15]. According to Rodriguez et al. [27], two main components, quercetin glycoside and quercetin di-glycoside are responsible for 80% of the total flavonoids in onions. The structural formula of quercetin contains all the structural components necessary for antioxidant and pro-oxidant activity [28]. Therefore, the coating’s potential for increasing b* color index can be explained by a possible antioxidant activity from these flavonoids present in edible onion coating. through the limitation of myoglobin oxidation and metmyoglobin accumulation, consequently leading to the control of lipid oxidation [19,21].

The edible onion film showed a higher chroma value than uncoated burger beef patties throughout storage, which may be appealing to consumers at purchase time. Fresh meat typically becomes less red and lighter after a few days. This may also be related to the fact that the coated burgers had less microorganism growth. The inhibition of microbial growth prevented excess oxidation thus, limited the reduction in a* [21]. Thus, an edible coating that can maintain redness and intensify the meat color could lead to an extension in burger patties color display-life [29].

The pH value of beef burger patties without application of edible onion film increased with storage time, probably as a result of the activity of microbial-based enzymes, which can degrade meat proteins into nitrogenous compounds, such as ammonia and trimethylamine [30,31] and thus, the pH value increases. The application of the edible onion film reduced the pH of the beef burger patties, especially between 3 and 6 days. This decrease can be attributed to a possible ability of the edible onion film to reduce permeability to carbon dioxide due to the likely presence of antibacterial agents, thus, helping, to reduce microbial growth and proliferation, which are reduced at lower pH [32]. An adequate decrease in pH is directly related to color, tenderness and the muscle’s ability to retain water [33].

The lower retention capacity and greater cooking loss at the end of storage at 9 days by the beef burger patties with edible onion film can be explained by the higher water content present in the treated samples due to the inclusion of the edible onion film itself, which has a hydrophilic nature, thus occurring a rapid moisture absorption, which diffuses through the material [16].

The non-heme iron content has capacity to catalyze lipid oxidation [34,35], thus justifying the decrease in heme iron content and increase values and increase in TBARS values, which is an indicator of secondary oxidation products such as malondialdehyde (MDA), which are formed during the reaction of lipid oxidation reaction [36,37]. The application of the onion edible film had no effect on the lipid peroxidation process. Adding antioxidants to meat products does not always have the desired effect. Green tea extracts and propolis extracts, known for their antioxidant activities, were not able to inhibit the lipid oxidation process when added to ground beef, and in some cases, depending on the concentration used, they presented pro-oxidant effects [38,39].

The application of the edible onion film in beef burger patties provided, throughout the storage period, less microbial growth and proliferation, demonstrated the antimicrobial potential of onion [40]. The high antimicrobial activity of onion peel residue extracts was observed against *Escherichia coli*, *Pseudomonas fluorescens*, *Bacillus cereus*, and *Aspergillus niger* fungi, *Trichoderma viride* and *Penicillium cyclopium* [37]. Behbahani and Imani [31] observed that edible coatings containing vegetable extracts have the potential to improve the microbial safety and shelf life of beef and this can occur by several mechanisms, including attacking the phospholipid bilayer of the cell membrane, interrupting enzymatic systems and damaging the genetic material of bacteria that, when they come into contact with the food surface, release the active compounds and, as a result, end up helping to inhibit/delay bacterial multiplication [41].

Processing storage time and cooking are the main factors that determine the sensory proprieties of foods treated with coating from natural sources [42]. In the present study, it was observed that the acceptance of the beef burger patties preserved with edible onion coating, regardless of the salt (75.8%). Only 2.5% of panelists did not mention preference for applying edible onion coating. Among the characteristics of edible coatings are the unique sensory properties when they are composed of fruits and vegetables that are desirable for applications in the food industry, as for sushi wraps, form pizza crust and toppings, or even coating for snacks in order to improve the nutritional quality and optimize the organoleptic characteristics of these foods [6,43]. In our study, the presence of lacristemic and flavoring compounds found specifically in the genus *Allium* may have been responsible for positively impacting the sensory characteristics of the beef burger patties. The improvement in color indexes, as well as in other physicochemical aspects, may be associated with a greater preference for beef burger patties preserved with edible onion coating [44,45], as color is a very important aspect in the presentation of bovine meat products and, without a doubt, is one of the points that most influence the purchase decision by consumers, considering that it is directly associated with food deterioration [46,47,48].

## 4. Materials and Methods

### 4.1. Ethical Considerations and Design Experimental

This experiment was conducted at the Federal University of Campina Grande (UFCG), Patos city, Brazil. All protocols were approved by the Research Ethics Committee on of the UFCG, with protocol n° CAAE: 45259321.0.0000.5182. The beef burger parties were randomly distributed in a completely randomized design distributed in a factorial design 2 × 4 with two treatments (use or no use of edible onion coatings) and four beef burger patties storage times (0, 3, 6 and 9) and three and ten replicates (only for texture evaluation as indicated) according to the realized analyses.

### 4.2. Obtaining, Handling and Applying Onion-Based Film

The films were produced and supplied by Dr. Diogenes dos’ Santos Dias from BioSmart Nanotechnology (BioSmart®, Araraquara, São Paulo, Brazil). The medium-sized onion (*Allium cepa* L.) bulbs were obtained at markets in the city of Araraquara, SP, Brazil (geographical coordinates: latitude: −21.7946; longitude: −48.1766 21° 47′ 41″ S, 48° 10′ 36″ W) [16]. Onion (*Allim cepa* L.) yellow-type and medium size (average moisture content of about 89%) were selected and separated for the production of edible films. Onion bulbs were previously washed with water to remove soil impurities during harvest and after transport. After that, the outer layers that were dry or deteriorated were removed before the bulbs were cut lengthwise into four pieces and washed again. The films were prepared by casting formulations comprising raw onion pulp and hydrothermally treated (1% by weight solids) according to the methodology described by Dias et al. [16] and Barreto et al. [17].

A total of 100 g raw pulp washed from the onion bulbs were placed in an industrial blender (FAK 800 W, 4 L, SIEHE Corporation, Shanghai, China), filtered through a qualitative filter paper (grade 292, Boeco qualitative filter; Sigma-Aldrich Corporation, San Luis, Missouri, EUA), washed with distilled water ten times to eliminate the characteristic odor. Then, a 0.5% aqueous suspension (*w*/*w*) was poured into 90 mm diameter Petri dishes (Kasvi, clear polystyrene; Sigma-Aldrich Corporation; San Luis, Missouri, EUA) and dried in an exhaust at room temperature with circulating air for 6 h or until detachment of the films.

### 4.3. Beef Burger Patties Preparation

The beef burger patties were prepared using the quadriceps femoris muscle following the method used by Ramos et al. [18]. In the processing, excess connective tissue was removed leaving only the muscle which was grounded to obtain a homogeneous meat. To obtain a homogeneous mass, the beef (81.3%) and the pork fat (15%) were weighed, manually mixed, ground in beef grinder ECCEL^®®^ (model MCIE-10, São Paulo, Brazil) using 8 mm discs and placed in plastic trays. To each homogeneous mass was added 30 g of salt. The mixing was carried out manually for 20 min and subsequently conditioned to a refrigerator for 12 h at 4 °C.

After the resting period, 100 g of the beef burger patties were molded in a manual press with a diameter of 9.5 cm (100 g). And then, the beef burger patties were individually packaged with edible onion films, labelled and cooled at 4 °C [18], for further analysis of shelf life (0, 3, 6 and 9). It is noteworthy that the salt was used only in the samples that were submitted to sensory analysis.

### 4.4. Physicochemical Analyses

The pH of beef burger patties was obtained immediately after the preparation, with a digital skewer type probe (Testo 205^®®^, São Paulo, Brazil), calibrated with a buffer solution with between pH 4.0 and pH 7.0 at temperature between 6–7 °C for 40 min.

Beef burger patties color indexes were evaluated Miltenburg et al. [49] at the referred times from the standardization of the cuts of beef burger in a thickness of 15 mm, followed by exposure to air for 30 min in a refrigerated environment (4 °C) so that the readings could then be taken with the aid of a colorimeter (Konica Minolta, model CR-400), operating in the CIELAB system (L*,a*,b*), where L* is the luminosity, varying from black (0%) to white (100%); a* the intensity of the red color, varying from green (–a) to red (+a); and b* the intensity of the yellow color, varying from blue (–b) to yellow (+b). The aperture port was with glass cover and the beef burger patties samples were measured using illuminant D65. This device was calibrated before each analysis with a standard white tile. Three measurements were taken at different points on the beef burger patties, using the mean values to represent the color. The color saturation index (Chroma, C*) determination used the equation described by Hunt and King [50]:

*C (chrome) = (a*^2^ + b*^2^)^0.5^ (1)

Water holding capacity (WHC) was determined as weight loss. It was used a 2.0 g beef burger sample, that was placed on a circular filter paper, between two acrylic plates, then a 3.4 kg equivalent force was put on the top of the paper for approximately 5 min. For each sample, results were expressed as a percentage of weight loss relative to its initial weight.

The analysis of cooking weight loss (CWL) followed American Meat Science Association [51] recommendations. The evaluations used two samples (2.5 cm thick), free of subcutaneous fat. The samples were placed on a grill (George Foreman Jumbo Grill GBZ6BW, Rio de Janeiro, Brazil) to cook, also was used a stainless-steel thermocouple (Gulterm 700; Gulton of Brazil) inserted in the beef burger to monitor the temperature of each steak until it reached 71 °C at the geometric center of the sample. Afterward, the steaks were removed from the cook apparat and then were exposed to room temperature to stabilization. The steaks were then weighed again. Finally, the CWL of each sample was obtained by the difference in weight of the samples (before and after cooking), with values described as percentage of exudate.

The TPA was determined in the cooked beef burger patties in a TA-XT plus texture analyzer (Stable Micro Systems, Godalming, England) and using its own Exponent Gram equipment, version v.51.1.0, based on the method described by Bourne [52]. The parameters performed were hardness (N), being the maximum force to compress the product, cohesion: which the sample can be deformed after rupture; springiness: the ability that the product tends to regain its original shape after the force is removed, and chewiness, i.e., the work required to chew the sample when swallowing. For TPA results, ten repetitions were measured for each treatment.

The total heme iron content was determined as previously described in the study by [53], where 2 g of each beef burger sample, in triplicate, was homogenized with 9 mL of acidified acetone (90% acetone, 8% deionized water, 2% HCl). Then, the homogenate was placed for 1 h at 25 °C in the dark and subsequently centrifuged for 10 min. The absorbance of the filtrate was determined at 640 nm. The amount of heme iron was expressed in µg/g of beef burger and calculated using the following equation:

Heme iron = A640 × 680 × 0.0882(2)

### 4.5. Lipid Oxidation of Beef Burger Patties

The lipid oxidation indicator was performed from thiobarbituric acid reactive substances (TBARS), according to the methodology of Witte et al. [54] in which the value of substances that react to TBA (TBARS) was calculated using a standard curve of malonaldehyde (MDA). For this, five grams of beef burger from each sample (triplicate) were mixed with 20 mL of trichloroacetic acid (5%), homogenized for 5 min and centrifuged at 12,000× *g* for 10 min. Then 4 mL of the supernatant was mixed with 4 mL of 0.02 M TBA and incubated in a water bath at 100 °C for 60 min. Absorbance was measured at 532 nm and the results expressed as µMoL of MDA/g of beef burger.

### 4.6. Microbiological Analysis of Beef Burger Patties

A total of 25 g of beef burger patties samples (in triplicate) with and without onion coating in all storage times were collected in sterilized bottles containing 225 mL of maximum recovery diluent (0.1% (*w*/*v*) of peptone in 0.9% NaCl solution (*w*/*v*)), and then homogenized for 5 min at each proposed shelf life. In a sterile environment, dilutions from 10^−1^ to 10^−5^ were made using the maximum recovery diluent. One milliliter of each dilution was added to sterile Petri dishes, then approximately 15 mL of Plate Count Agar culture medium at 40 °C was added. The plates were carefully shaken and incubated at 30 °C and 4 °C for 3 days and 7 days, respectively, for the counting of mesophilic and psychrophilic microorganisms [55].

### 4.7. Sensory Attributes

For the sensory evaluation, the beef burger patties with edible onion films applied were divided into 4 treatments from the preparation of the burger: treatment 1—with edible onion films/with salt; treatment 2—with edible onion films/no salt; treatment 3—without edible onion coating/with salt; and treatment 4—without edible onion films/no salt. This was realized to test the action of the edible onion film as a flavoring as it is a widely used condiment in food preparation,

Then, consumer appeal was assessed using a panel made up of 80 untrained tasters, including 40 women and 40 men (aged between 18 and 54 years) [48]. Two beef burger patties samples of each treatment were cut into cubes (approximately 3–5 g) and grilled on a preheated electric grill (George Foreman Jumbo Grill GBZ6BW, Rio de Janeiro, Brazil) at 170 °C until the temperature of the geometric center reached 71 °C. The beef burger patties samples were transferred to preheated coded beakers covered with aluminum foil to guarantee the minimum loss of heat and volatile aroma, and these were kept in a water bath inside a glass beaker (Thermomix^®®^, São Paulo, Brazil) at 75 °C only that the temperature of the samples remains between 65 and 70 °C until distribution to the tasters [51].

Sensory evaluations were performed in 10 individual cabins on a single day between 09:00 and 11:00 a.m., with 10 participants per session (*n* = 8). The sessions consisted of eight tasting rounds of booths at pre-established times. Each taster received eight samples of beef burger (four treatments in duplicate), randomly distributed and coded with three numerical digits [48]. The duration time of each session was approximately 15 min. Consumers were directed to individual booths in the laboratory, after which samples, water-and-salt biscuits and water were delivered, where the last two items were provided to cleanse any residual flavor between samples. The attributes of taste, odor, texture (tenderness), appearance and overall acceptance of beef burger patties were evaluated, and these attributes were chosen through the 9 points hedonic scale, ranging between: “1 disliked it extremely” and “9 liked it extremely” on forms delivered at the beginning of the evaluation. Panelists were also asked to rank the beef burger patties according to their preference.

### 4.8. Statistical Analysis

The beef burger patties were randomly distributed in a completely randomized design distributed in a factorial design 2 × 4 with two treatments and four beef burger patties storage times (0, 3, 6 and 9 days) and the replicates according to analyses realized. The following statistical model was used:

Yijk = u + Oi + Tj + (OT)ij + Eijk(3)

where: u is the overall mean, Oi is the effect of adding edible onion film, Tj is the effect of shelf life or storage, (OT)ij is the interaction between edible onion film and shelf life, and Eijk is random error.

For sensory characteristics, the linear mixed model was used to analyze taste, odor, texture (tenderness), appearance and overall acceptance of each sample to identify the factors influencing the response. The fixed effects in the model included the use of edible onion film with or without salt as a condiment, and the random effects in the model included panelists and sessions.

Results were expressed as mean ± standard deviation. Data were statistically evaluated using the GraphPad Prisma Software. The means of two of the groups were compared using Student’s *t* test. To compare three or more groups, one-way analysis of variance (ANOVA) was used, followed by Tukey using the GraphPad Prisma Software. (GraphPad Prism®, São Paulo, Brazil). Sensory perception was analyzed using Fisher’s LSD test and preference classification was expressed using the GraphPad Prisma software. Statistical data were considered significant when *p* < 0.05.

## 5. Conclusions

The edible onion film is recommended for preserving beef burgers patties, as it delays the proliferation of unwanted microorganisms, stabilizing and improving the color parameters and thus improving sensory attributes of beef burger patties and being more accepted by the consumer. This edible film may be used in the meat and meat product packaging industry, replacing conventional packaging, ensuring improved quality and increased shelf life of food.

## Figures and Tables

**Table 1:** Coloration parameters of beef burger patties uncoated (control) and coated with edible onion film submitted to different storage factor (4 °C).

Variables	Treatment	Storage Times (Days)			
		0 Days	3 Days	6 Days	9 Days
Luminosity (L*)	Control	44.22 ± 0.59 ^Aa^	43.02 ± 0.75 ^Ba^	42.35 ± 0.44 ^BCa^	41.24 ± 1.02 ^Ca^
	Edible onion film	42.97 ± 0,29 ^Ab^	40.83 ± 1.42 ^BCb^	42.18 ± 0.80 ^ABa^	39.81 ± 0.72 ^Cb^
Redness (a*)	Control	14.38 ± 0.84 ^Ab^	11.58 ± 0.87 ^Bb^	9.71 ± 0.59 ^Cb^	6.00 ± 0.25 ^Db^
	Edible onion film	16.02 ± 0.86 ^Aa^	14.42 ± 0.73 ^Ba^	14.42 ± 1.10 ^Ba^	13.03 ± 0.77 ^Ba^
Yellowness (b*)	Control	7.35 ± 0.25 ^Ab^	7.13 ± 1.05 ^Aa^	5.04 ± 0.47 ^Bb^	3.77 ± 0.79 ^Cb^
	Edible onion film	9.39 ± 0.40 ^Aa^	8.44 ± 0.82 ^Bb^	7.99 ± 0.25 ^Ba^	8.75 ± 0.35 ^ABa^
Chrome (C*)	Control	16.16 ± 0.72 ^Ab^	13.62 ± 1.11 ^Bb^	10.95 ± 0.66 ^Cb^	7.13 ± 0.25 ^Db^
	Edible onion film	18.53 ± 0.91 ^Aa^	16.72 ± 0.91 ^Ba^	16.49 ± 0.98 ^Ba^	15.69 ± 0.82 ^Ba^

Values described as mean ± standard deviation (*n* = 3). Different uppercase letters in the same line indicate statistically significant differences between storage times (days) for each treatment by the Tukey test (*p* < 0.05). Different lowercase letters in the same column indicate statistically significant differences between coated and uncoated beef burger patties by the Student’s t-test (*p* < 0.05).

**Table 2:** Physicochemical parameters and lipid oxidation of beef burger patties uncoated (control) and coated with edible onion performed at different storage factor (4 °C).

Variables	Treatment	Storage Times (Days)			
		0 Days	3 Days	6 Days	9 Days
pH	Control	5.68 ± 0.39 ^aB^	6.11 ± 0.10 ^aAB^	6.19 ± 0.04 ^aA^	5.20 ± 0.11 ^aC^
	Edible onion film	5.79 ± 0.07 ^aA^	5.59 ± 0.05 ^bB^	5.41 ± 0.04 ^bC^	4.40 ± 0.06 ^bD^
WHC ^1^ (%)	Control	27.43 ± 0.85 ^aA^	22.03 ± 2.20 ^aB^	22.43 ± 1.53 ^aB^	16.05 ± 0.66 ^aC^
	Edible onion film	20.06 ± 2.84 ^bAB^	17.75 ± 2.85 ^aAB^	23.31 ± 1.84 ^aA^	16.01 ± 2.41 ^aB^
Cooking loss (%)	Control	21.60 ± 3.64 ^aB^	15.94 ± 2.38 ^aB^	19.70 ± 1.99 ^aB^	23.73 ± 3.36 ^aA^
	Edible onion film	16.28 ± 3.94 ^aB^	16.75 ± 2.03 ^aB^	17.86 ± 1.93 ^aB^	26.97 ± 0.27 ^aA^
Iron content (μg/g)	Control	20.75 ± 1.58 ^aA^	16.85 ± 0.86 ^aB^	15.29 ± 1.78 ^aB^	14.27 ± 0.72 ^aB^
	Edible onion film	18.79 ± 0.30 ^aA^	18.17 ± 1.02 ^aA^	16.21 ± 1.66 ^aB^	14.09 ± 0.76 ^aB^
Lipid oxidation ^2^	Control	0.78 ± 0.06 ^aC^	1.10 ± 0.12 ^aC^	1.72 ± 0.10 ^aB^	2.12 ± 0.23 ^aA^
	Edible onion film	0.80 ± 0.07 ^aC^	1.07 ± 0.01 ^aC^	1.67 ± 0.19 ^aB^	2.23 ± 0.20 ^aA^

Values described as mean ± standard deviation (*n* = 3). Different uppercase letters in the same line indicate statistically significant differences between storage times (days) for each treatment by the Tukey test (*p* < 0.05). Different lowercase letters in the same column indicate statistically significant differences between coated and uncoated beef burger patties by the Student’s *t*-test (*p* < 0.05). ^1^ Water holding capacity (WHC); ^2^ Expressed in µMoL malondialdehyde/kg beef burger patties.

**Table 3:** Texture profile of beef burger patties uncoated (control) and coated with edible onion performed at different storage factor (4 °C).

Texture Parameter	Treatment	Storage Times (Days)			
		0 Days	3 Days	6 Days	9 Days
Hardness (N)	Control	17.35 ± 3.54 ^aB^	15.83 ± 4.27 ^aB^	14.87 ± 2.56 ^aB^	20.09 ± 3.40 ^aA^
	Edible onion film	16.26 ± 2.90 ^aB^	23.97 ± 6.13 ^aA^	15.15 ± 3.90 ^aB^	20.27 ± 1.92 ^aA^
Springiness	Control	0.75 ± 0.09 ^aA^	0.85 ± 0.01 ^aB^	0.85 ± 0.05 ^aB^	0.89 ± 0.02 ^aB^
	Edible onion film	0.86 ± 0.01 ^aA^	0.83 ± 0.10 ^aA^	0.81 ± 0.02 ^aA^	0.85 ± 0.01 ^bA^
Chewability (N)	Control	850.02 ± 92.21 ^aB^	590.96 ± 5 0.79 ^bC^	689.07 ± 61.84 ^aC^	1060.51 ± 75.85 ^aA^
	Edible onion film	688.41 ± 42.89 ^bB^	965.95 ± 87.28 ^aA^	693.96 ± 38.87 ^aB^	762.48 ± 71.00 ^bB^
Cohesiveness	Control	0.50 ± 0.05 ^aA^	0.42 ± 0.05 ^aA^	0.45 ± 0.07 ^aA^	0.51 ± 0.05 ^aA^
	Edible onion film	0.50 ± 0.03 ^aA^	0.46 ± 0.05 ^aB^	0.39 ± 0.07 ^aB^	0.44 ± 0.05 ^aA^

Values described as mean ± standard deviation (*n* = 10). Different uppercase letters in the same line indicate statistically significant differences between storage times (days) for each treatment by the Tukey test (*p* < 0.05). Different lowercase letters in the same column indicate statistically significant differences between coated and uncoated beef burger patties by the Student’s *t*-test (*p* < 0.05). (N = Newton).

## Data Availability

The data that support the findings of this study are available from the corresponding author upon reasonable request.

## Sample Availability

Not available.
